# Improved clinical outcomes after revision arthroplasty with a hinged implant for severely stiff total knee arthroplasty

**DOI:** 10.1007/s00167-018-5235-5

**Published:** 2018-10-26

**Authors:** Paul J. H. van Rensch, Petra J. C. Heesterbeek, Gerjon Hannink, Gijs G. van Hellemondt, Ate B. Wymenga

**Affiliations:** 1 Sint Maartenskliniek, PO Box 9011, 6500 GM Nijmegen, The Netherlands; 2 Department of Orthopedics, Radboud University Medical Center, PO Box 9101, 6500 HB Nijmegen, The Netherlands

**Keywords:** TKR, Revision total knee arthroplasty, Hinged total knee arthroplasty, Stiffness, Arthrofibrosis, Range of motion

## Abstract

**Purpose:**

Management of the severely stiff total knee arthroplasty (TKA) is challenging, with the outcome of revision arthroplasty being inferior compared to the outcome for other indications. The aim of this study was to analyse the outcome after revision TKA with hinged-type implants for severely stiff TKA [range of motion (ROM) ≤ 70°] at 2 years.

**Methods:**

A cohort of 38 patients with a hinged-type revision TKA (Waldemar Link or RT-Plus) and preoperative ROM ≤ 70° were selected from a prospectively collected database. ROM, visual analogue scale (VAS) for pain and satisfaction and Knee Society Score (KSS) were obtained preoperatively and at 3 months, 1 year and 2 years. Pre- and postoperative outcome were compared at 2 years.

**Results:**

There was a significant increase in ROM and KSS. VAS pain scores did not differ significantly. The median ROM at 2 years was 90° (range 50°–125°) with a median gain of 45° (range 5°–105°). Median VAS pain was 28.5 (range 0–96) points and median VAS satisfaction was 72 (range 0–100) points at 2 years. Twelve patients suffered a complication. Recurrent stiff knee was the most frequently reported complication (*n* = 5).

**Conclusions:**

Hinged-type revision TKA following a severely stiff TKA renders a significant, although moderate, clinical improvement at 2 years.

**Level of evidence:**

Retrospective case series. Level IV.

## Introduction

Stiffness following total knee arthroplasty (TKA) is a challenging problem in orthopaedic surgery. When looking at revision TKA, it has been shown that patients who were revised for severely stiff TKA have the worst outcome directly postoperatively and remain worse at 2 years with respect to range of motion (ROM), pain and satisfaction score, and Knee Society Score (KSS) when compared to other indications (revision for septic loosening, aseptic loosening, component malposition or instability) [1]. Analysis of the outcome and treatment of severely stiff TKAs has proven to be challenging, mostly because the aetiology is largely unknown and fairly diverse. Furthermore, comparing results reported in literature is complicated by variable definitions of stiffness that are being used [2–4].

Management of a severely stiff TKA consists of physiotherapy, manipulation under anaesthesia (MUA), arthroscopic debridement or open debridement [5–10]. Revision arthroplasty is most commonly reserved for the correction of technical errors in the severely stiff TKA, such as malrotation, malpositioning and instability [8, 9, 11].

In an attempt to further improve the outcome, a series of patients with severely stiff TKA was treated with a hinged-type revision TKA. Hereby, a more extensive soft tissue release was enabled without the risk of causing instability [12]. Many authors have looked at the results of revision TKA using a condylar implant on the outcome of severely stiff TKA [2, 8, 13–17]. According to Cohen et al., revision TKA, although being a viable option for some patients, still does not offer a solution for all patients suffering from a severely stiff TKA. Farid et al. are the only ones that partly looked at the effect of radical adhesiolysis and revision TKA using a hinged-type TKA [14]. The aim of this study was to analyse the outcome of revision for severely stiff TKA using a hinged-type TKA system. It was hypothesized that revision of severely stiff TKA using a hinged-type implant leads to a significant increase in ROM, VAS satisfaction, and KSS scores, and a significant decrease in pain at 2 years follow-up.

## Materials and methods

Patients were retrospectively selected from a prospectively collected data set, as previously described by Van Kempen et al. [1]. Patients were selected from the database for the present analysis when they had received a hinged-type revision TKA because of a severely stiff TKA in the period between June 2004 and December 2012. All cases were primary TKA following osteoarthritis.

In this study, a severely stiff TKA was defined as a ROM < 70°, according to the International Consensus of the definition and classification of fibrosis [18]. All revisions were performed by two experienced orthopaedic knee surgeons at our institution. Patients with a revision due to periprosthetic joint infection or with a follow-up of less than 1 year were excluded from the analysis.

The used hinged implants were the Waldemar Link Endo-Modell^®^ (Link, Hamburg, Germany) (*n* = 7) or the RT-Plus (Smith & Nephew, Memphis, TN, USA) (*n* = 31). Both implants were rotating hinge TKA. Choice of implants was based on the surgeon’s preference. All patients in the database were evaluated preoperatively (pre-revision), perioperatively, at 3 months, and at 1 and 2 years postoperatively. All evaluations were done during routine follow-up visits.

During all procedures, a rigorous debridement of fibrous tissue and extensive release of the joint capsule were performed. Six tissue cultures were routinely taken to evaluate for periprosthetic joint infection.

**Fig. 1:**
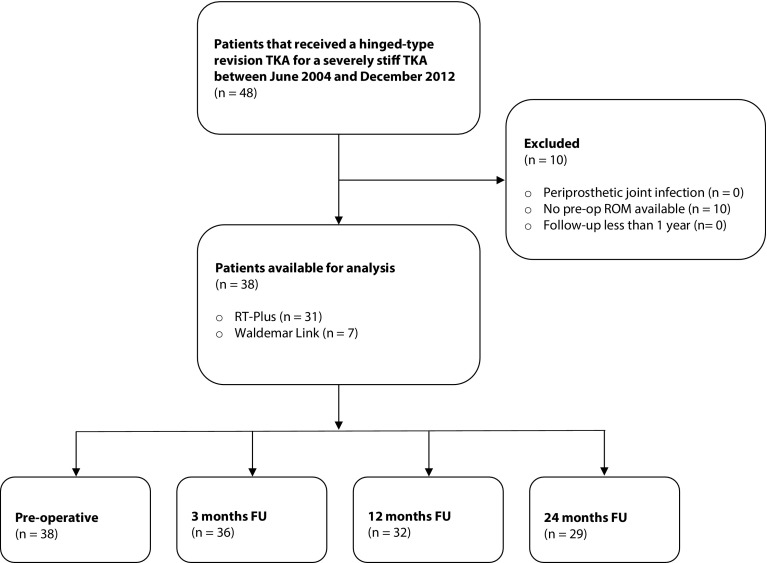
Flowchart

In total, the data of 38 patients were available for analysis (Fig. 1; Table 1). In all patients, a detailed and personalized workup was performed to identify the cause of stiffness. This workup contained a standard antero-posterior, lateral, and patellar skyline, and a standing full-leg radiograph to assess alignment. Depending on patient characteristics, additional tests were performed. When malpositioning or aseptic loosening was suspected, a CT scan was performed to determine the rotation of the components and to assess bone loss. Malrotation was measured according to Berger et al. and Victor et al. [19, 20]. The presence of an infection was evaluated according to the Musculoskeletal Infection Society (MSIS) criteria [21], including blood samples (CRP, ESR and WBC) and/or an aspirate of the joint fluid (culture and WBC count/differentiation). Additional stress radiographs were performed in case of suspected instability.

**Table 1:** Patient demographics

Age (years) [mean (range)]	64 (40–85)
Gender (male:female)	12:26
Side (right:left)	24:14
Preop ROM [median (range)]	50° (5°–70°)
Underlying indication	
Malposition	15
Aseptic loosening	7
Instability	2
Stiffness e.c.i	14

## Outcome

The outcome measurements that were collected in this database consisted of the Knee Society Scoring System (KSS) (assessed by the orthopaedic surgeon or resident in the outpatient clinic) and 100-mm visual analogue scales (VAS) for both pain and satisfaction (scored by the patients; 0 is no pain and 100 the worst pain imaginable, 0 is very dissatisfied and 100 very satisfied respectively). Complications were defined as any type of adverse events related to functioning of the revision implant, warranting significant additional (non)surgical treatment.

Approval of this study was given by the hospital’s investigational review board. The Medical Ethical Review Board granted a waiver for this study (ID: 2003/173).

### Statistical analysis

Descriptive statistics [median (range)] were used to quantify clinical outcome. Wilcoxon’s signed rank tests were used to compare the preoperative with postoperative values at 2 years. Statistical analysis was performed using STATA 13 (StataCorp, College Station, TX, USA). The level of statistical significance was set at *p* < 0.05.

## Results

In 14 of the 38 patients, analysis yielded no underlying reason for arthrofibrosis. Malpositioning was the most common concurrent finding, followed by loosening and instability (Table 1).

The range of motion significantly increased from a median of 50° (5°–70°) preoperatively to a median of 90° (50°–125°) at 2 years (*p* < 0.0001) (Table 2). At 2 years, for nine patients no data were available as these patients terminated routine follow-up. Six out of the remaining 29 patients had an ROM of less than 70°, 6 had an ROM of 70°–89° and 17 showed an ROM of ≥ 90°. The KSS clinical and KSS functional showed a significant increase at 2 years postoperatively, and VAS pain did not improve significantly at 2 years (Table 3).

**Table 2:** Gain in ROM (°) from the KSS

3 months postoperatively	40° (15°–120°) [*N* = 36]
1 year postoperatively	40° (10°–90°) [*N* = 32]
2 years postoperatively	45° (5°–105°) [*N* = 29]

Values are median (range)

**Table 3:** Outcomes, values are median (range)

Outcomes	Preop (*N* = 38)	3 months (*N* = 36)	12 months (*N* = 32)	24 months (*N* = 29)	*p* value*
KSS					
ROM	50° (5°–70°)	90° (50°–125°)	90° (30°–125°)	90° (50°–125°)	< 0.0001
Clinical	43 (4–89)	65 (32–100)	72 (25–97)	76 (10–100)	< 0.001
Function	30 (5–70)	50 (5–100)	70 (30–100)	60 (5–100)	< 0.05
VAS					
Pain	62.5 (0–100)	33 (0–100)	23 (0–81)	28.5 (0–96)	n.s.^b^
Satisfaction	NA	74 (3–100)	78.5 (6–100)	72 (0–100)	NA
Question					
Yes:no	NA	27:7^a^	25:7	23:6	NA

*p values are at 2 years postoperatively, compared to preoperatively

^a^Not all patients answered the questions at 3 months

^b^Not significant

VAS satisfaction was fairly constant (Table 3). Additionally, patients were asked if they would undergo the same procedure again. At 2 years, this question was answered positively by 23 out of 29 patients (79%).

Twelve of 38 patients suffered a complication (Table 4). Recurrent stiff knee was the most frequent complication (five patients, one of whom also had a pulmonary embolism). This was treated with MUA in one patient and with a lateral release in one other patient. The other three patients had late postoperative recurrent stiff knee, for which an expectative treatment was chosen. One patient had persistent pain without satisfactory explanation, for which the patient was referred to our pain clinic. Aseptic loosening occurred in two patients, one case of tibial aseptic loosening and one case of femoral aseptic loosening. Prosthetic joint infection was seen in one patient, eventually resulting in amputation after earlier unsuccessful debridement, antibiotics and implant retention (DAIR), implant removal and re-implantation. One patient died within 1 year of the operation, unrelated to the operation or a complication thereof.

**Table 4:** Complications

Complication	No. of cases	Treatment
Osteonecrosis tibia	1	ORIF^b^ + solid bone graft (20 months)
Early infection	1	DAIR^c^, explantation, re-implantation, eventually amputation (5 years)
Extension lag (40°)	1	Arthrodesis (3 years)
Recurrent arthrofibrosis	5	MUA in 1 patient (5 months), lateral release in 1 patient (6 months)
Pulmonary embolism^a^	1	Antithrombotic therapy
Aseptic loosening tibial component	1	Re-revision (1.5 year)
Aseptic loosening femoral component	1	Re-revision (2.5 year)
Persistent pain	1	Pain clinic

^a^Additional complication with recurrent arthrofibrosis in the same patient

^b^Open reduction with internal fixation

^c^Debridement, antibiotics and implant retention

## Discussion

The most important finding of this study was that a hinged implant significantly improved ROM and KSS clinical and functional scores in patients with a severely stiff knee arthroplasty at 2 years postoperatively. With respect to VAS pain, no significant improvement was seen, due to the large spread in reported pain. The present study presents the largest cohort following a hinged-type revision TKA for severely stiff TKA.

Knee flexion is essential for mobility, for recreational activities as well as for activities of daily living (ADL). A decrease in knee ROM can therefore limit a patient’s ability to perform ADL tasks. When looking at ROM related to ADL, patients require an average of 83° knee flexion to climb stairs foot over foot. To sit in a chair without using one’s hands requires, on average, 93° knee flexion. Tying one’s shoes while seated requires an average of 106° flexion [22]. Riding a bicycle requires, on average, 100°–110° of knee flexion (90° with modifications to the bike).

So, even a mild increase in knee flexion can make the difference between walking and being able to ride a bike, which is very important for mobility and quality of life.

Therefore, even while the effects of a revision with a hinged TKA seem moderate, these results are relevant to our patients.

With respect to the question if the patient would undergo the same procedure again, we looked in more detail at the patients who changed their answer from ‘Yes’ to ‘No’ somewhere during the follow-up period to see if this was related to complications. Seven patients changed their answer in the follow-up period from ‘Yes’ to ‘No’: of them, one patient had unexplained pain and three patients showed recurrent stiffness. The other three patients reported unmet expectations with respect to ROM and improvement in ADL. This shows that counseling on expectations remains an important part of the consultation in patients with a severely stiff knee following TKA.

The gain in ROM found in this study is consistent with the findings reported by other authors, given the right timing of intervention [2, 13, 17, 22, 23]. So although a thorough excision of the fibrous tissue is performed, revision arthroplasty using a hinged-type TKA is not the answer for all patients with a severely stiff TKA. This underlines the importance of finding and better understanding the aetiology of arthrofibrosis. In a recent paper, Clement et al. found that male gender, lung disease, diabetes, back pain, and preoperative stiffness rendered an increased risk for developing a severely stiff TKA [24]. This is an important step in better understanding the development of arthrofibrosis and can help in counselling patients when considering a primary TKA or a revision for severely stiff TKA. Future research, however, should be directed at finding the biological basis for arthrofibrosis.

Some potential limitations have to be discussed. First, two different implants were used which might have influenced the outcomes. However, because the treatment of the arthrofibrosis was a radical excision of fibrous tissue and soft tissue release, the outcome was not thought to be influenced by implant design. Furthermore, both implants had a rotating hinge design. Second, there is the risk of selection bias. Typically, hinged prostheses were used in the worst cases, but selection of implant type was done by the surgeon, and not by randomization. Third, because of the nature of data collection (during standard follow-up visits), there were some missing data. In patients with missing data, scores of the previous visit were evaluated. Of the nine patients with missing data for ROM, two patients had an ROM of 85° at the previous visit, three patients had an ROM of 100°, two an ROM of 110°, and two an ROM of 120°. None of the patients with missing data for ROM had a recurrent severely stiff TKA at previous visits. Out of the seven patients with missing data for patient satisfaction, five indicated they would undergo the same operation again at the previous visit. Most patients indicated that they terminated further follow-up due to other issues (travel distance to clinic or general health issues).

Arthrofibrosis following TKA remains challenging for both patient and surgeon, especially in recurrent or late severely stiff TKA, where MUA and arthrolysis are not advocated [10]. The present study shows that revision with a Hinged-type TKA is a viable option for improving ROM and clinical outcome for these patients.

## Conclusion

Hinged-type TKA significantly improves ROM and KSS clinical and functional scores in patients suffering from a severely stiff knee arthroplasty 2 years after revision surgery.
